# Developmental Regulation of Sialoadhesin (Sheep Erythrocyte Receptor), a Macrophage-Cell Interaction Molecule Expressed in Lymphohemopoietic Tissues

**DOI:** 10.1155/1992/74945

**Published:** 1992

**Authors:** Lynn Morris, Paul R. Crocker, Maxine Hill, Siamon Gordon

**Affiliations:** 1 Walter and Eliza Hall Institute Melbourne Australia; 2 Pasteur Institute Paris France; 3 Sir William Dunn School of Pathology University of Oxford, South Parks Road Oxford OX1 3RE United Kingdom

**Keywords:** Sialoadhesin, macrophages, cell interaction, ontogeny

## Abstract

Stromal macrophages in lymphohemopoietic tissues express novel macrophagerestricted plasma membrane receptors involved in nonphagocytic interactions with other hemopoietic cells. One such receptor with lectinlike specificity for sialylated glycoconjugates on sheep erythrocytes and murine hemopoietic cells has been characterized immunochemically and termed sialoadhesin. We have examined sialoadhesin expression during mouse development to learn more about its regulation and function. Immunocytochemical, rosetting, and Western blot studies show that sialoadhesin is first detected on fetal liver macrophages on day 18 of development, 7 days after numerous F4/80^+^  macrophages are found within erythroblastic islands. In spleen and bone marrow, sialoadhesin appears between day 18 and birth, in parallel with myeloid development. Strongly labeled macrophages in the marginal zone of spleen, characteristic of adult lymphoid tissues, appeared gradually between 1–4 Isolation of fetal liver macrophages at day 14 confirmed that sialoadhesin was not involved in the binding of erythroblasts, which is mediated by a distinct cationdependent receptor (Morris et al., 1988, p. 649). Sialoadhesin could be expressed by isolated fetal liver macrophages after cultivation in adult mouse serum, a known source of inducer activity, but was not dependent on the presence of this inducer, unlike adultderived madrophages. Fetal plasma contained inducing activity on day 13, but adult levels were not reached until 2 weeks postnatally. These studies show that sialoadhesin is differentially regulated compared with the erythroblast receptor and F4/80 antigen, that it is not required for fetal erythropoiesis, and that its induction on stromal macrophages is delayed until the onset of myeloid and lymphoid development. Sialoadhesin provides a marker to study maturation and functions of macrophages during ontogeny of the lymphohemopoietic system.


*Developmental Immunology*, 1992, Vol. 2, pp. 7–17
Reprints available directly from the publisher
Photocopying permitted by license only

© 1992 Harwood Academic Publishers GmbH
Printed in the United Kingdom

### Developmental Regulation of Sialoadhesin (Sheep Erythrocyte Receptor), a Macrophage-Cell Interaction Molecule Expressed in Lymphohemopoietic Tissues

LYNN MORRIS†, PAUL R. CROCKER‡, MAXINE HILL§, and SIAMON GORDON§*

†*Walter and Eliza Hall Institute, Melbourne, Australia*
‡*Pasteur Institute, Paris, France*
§*Sir William Dunn School of Pathology, University of Oxford, South Parks Road, Oxford OX1 3RE, United Kingdom*

Stromal macrophages in lymphohemopoietic tissues express novel macrophage-restricted plasma membrane receptors involved in nonphagocytic interactions with other hemopoietic cells. One such receptor with lectinlike specificity for sialylated glycoconjugates on sheep erythrocytes and murine hemopoietic cells has been characterized immunochemically and termed sialoadhesin. We have examined sialoadhesin expression during mouse development to learn more about its regulation and function. Immunocytochemical, rosetting, and Western blot studies show that sialoadhesin is first detected on fetal liver macrophages on day 18 of development, 7 days after numerous F4/80$^{+}$ macrophages are found within erythroblastic islands. In spleen and bone marrow, sialoadhesin appears between day 18 and birth, in parallel with myeloid development. Strongly labeled macrophages in the marginal zone of spleen, characteristic of adult lymphoid tissues, appeared gradually between 1–4 weeks after birth, as the white pulp became enlarged.

Isolation of fetal liver macrophages at day 14 confirmed that sialoadhesin was not involved in the binding of erythroblasts, which is mediated by a distinct cation-dependent receptor (Morris et al., 1988, p. 649). Sialoadhesin could be expressed by isolated fetal liver macrophages after cultivation in adult mouse serum, a known source of inducer activity, but was not dependent on the presence of this inducer, unlike adult-derived macrophages. Fetal plasma contained inducing activity on day 13, but adult levels were not reached until 2 weeks postnatally. These studies show that sialoadhesin is differentially regulated compared with the erythroblast receptor and F4/80 antigen, that it is not required for fetal erythropoiesis, and that its induction on stromal macrophages is delayed until the onset of myeloid and lymphoid development. Sialoadhesin provides a marker to study maturation and functions of macrophages during ontogeny of the lymphohemopoietic system.

KEYWORDS: Sialoadhesin, macrophages, cell interaction, ontogeny.

### INTRODUCTION

Stromal macrophages (MØ) in hemopoietic organs form intimate cellular associations with developing blood cells and may influence their growth and differentiation (Crocker and Gordon, 1986; Crocker et al, 1988b). Two novel MØ-restricted hemopoietic cell-adhesion receptors have been identified in MØ isolated from murine tissues. The best characterized is a lectinlike hemagglutinin, first described on adult resident bone marrow MØ (Crocker and Gordon, 1986), that binds sheep erythrocytes (SE) via recognition of sialylated glycoconjugates. When expressed at high levels, this receptor, originally termed SER and now sialoadhesin (Crocker et al., 1991), mediates sialic acid-dependent attachment of murine myeloid and erythroid cells to murine MØ (Morris, Crocker, et al., 1991. P.R. Crocker, unpublished). The receptor is also present on specialized MØ populations in spleen, lymph nodes, and liver, but is expressed at low levels on certain other MØ populations defined by the F4/80 differentiation antigen (Austyn and

*Corresponding author.

Gordon, 1981). Sialoadhesin is regulated by a species-restricted plasma/serum factor required for maintenance of the receptor on isolated stromal MØ and for its induction on nonstromal MØ such as peritoneal cells (Crocker and Gordon, 1988a). An inhibitory mAb (SER-4) that defines a plasma membrane polypeptide of 185 kD has been used to purify sialoadhesin from spleen and label stromal MØ subpopulations by immunocytochemistry in adult lymphohemopoietic tissues (Crocker and Gordon, 1989).

A distinct hemopoietic cell-adhesion receptor that binds murine erythroblasts (Eb) by a divalent cation-dependent interaction (Morris et al., 1988) has been identified on fetal liver MØ (FLMØ), but this receptor has not been defined immunochemically. Recently, we have shown that a similar divalent cation-dependent receptor is present on adult bone marrow stromal MØ (Morris et al., 1991a). Although both sialoadhesin and the EbR contribute to binding of hemopoietic cells to MØ, the nature of the ligand-bearing cells and function of each receptor are unknown. Two recent observations with the SER-4 mAb implicate sialoadhesin in specific lymphohemopoietic cell interactions in situ. In bone marrow, sialoadhesin is concentrated at sites of contact with developing myeloid, but not erythroid cells (Crocker et al., 1990), whereas in spleen, high levels are expressed by marginal metallophils, a specialized subpopulation of MØ in contact with cells of the white pulp, and by MØ in the subcapsular sinus of lymph nodes (Crocker and Gordon, 1989) indicating a possible role in lymphoid-cell interactions with specialized MØ.

The ontogeny of the murine lymphohemopoietic system provides a further experimental model to learn about expression and function of sialoadhesin. Erythroid, myeloid, and lymphoid cells develop in a distinct sequence in different organs (Metcalf and Moore, 1971) and MØ defined by the plasma membrane antigen F4/80 are present at all sites of hemopoiesis (Morris, Graham, and Gordon, 1991b). We therefore used specific mAb to examine expression of sialoadhesin on MØ in fetal and newborn lymphohemopoietic tissues. We show that sialoadhesin appears relatively late in development; its expression prior to birth is better correlated with the onset of myelopoiesis rather than erythropoiesis, and postnatally with maturation of peripheral lymphoid organs. Induction of sialoadhesin is independent of that of F4/80, and may contribute to ontogeny of myeloid- and lymphoid-cell populations during development.

### RESULTS

### Distribution of Sialoadhesin During Development

In the fetus, hemopoietic activity occurs first in the yolk sac and sequentially in liver, spleen, and bone marrow. We have shown previously by immunohistochemistry using the MØ-specific F4/80 mAb that MØ are present in all these sites (Morris, Graham and Gordon, 1991b). In this study, we examined expression of sialoadhesin by MØ at different stages of development, using a specific rabbit polyclonal antiserum raised against the purified receptor (Crocker et al., 1991). This reagent blocks sialoadhesin rosetting activity, immunoprecipitates the same 185-kD molecule as SER-4 mAb and detects multiple epitopes on sialoadhesin. A MØ-specific rabbit polyclonal anti-F4/80 antiserum was used for comparison.

Sialoadhesin was not detected in yolk sac during development, although staining with the F4/80 antiserum showed that MØ were present from day 10 (d10) onwards (not shown). In fetal liver, strongly labeled, F4/80$^+$ stellate MØ were present by d14, but these did not stain with the sialoadhesin antiserum until d18 (Figs. 1A, 1B, and 1C). Initial plasma membrane labeling was patchy and staining intensity increased by birth (Fig. 1D). The distribution of labeled cells was identical to that of F4/80$^+$ stromal MØ within clusters that contained mainly erythroid and occasional myeloid cells. Stellate MØ, but not developing monocytes, were labeled by sialoadhesin antiserum. Although staining was restricted to MØ, it was less intense than that of F4/80 at all stages. Sinus-lining MØ resembling Kupffer cells continued to express sialoadhesin as hemopoiesis in liver declined postnatally (not shown).

In spleen, F4/80$^+$ MØ present by d17 did not express sialoadhesin (Fig. 2A vs Fig. 2B) until faintly labeled cells appeared on d18 (not shown). Numerous granulocytic cells were seen throughout spleen at this stage as were an unidentified population of large, round cells with peroxidase-positive granules (Fig. 2A). These cells lacked both MØ markers and disappeared 2 weeks after birth. Well-spread MØ expressing sialoadhesin were readily detectable throughout the red pulp 1 week after birth (Fig. 2C). Labeling was patchy and less intense than for F4/80, but defined the same red pulp MØ population. Early stages of developing white pulp contained no cells labeled with either ab. The characteristic, intensely sialoadhesin-bearing metallophil MØ found within the marginal zone of adult spleen (Crocker and Gordon, 1989) appeared during the following 3 weeks (Figs. 2E to 2H). Labeled cells at first were sparsely distributed, then formed a ringlike network of two cell layers within the marginal zone as the white pulp increased in size.

In bone marrow, sialoadhesin was expressed late in development. F4/80$^+$ MØ were present from d17, whereas sialoadhesin labeling was faint on d19, although readily detected after birth when myelopoiesis was evident. Neonatal thymus was also examined, but in contrast to F4/80, it contained only traces of sialoadhesin (not shown).

In all tissues examined, therefore, the sialoadhesin ag was restricted to selected MØ populations and expressed later in development than the F4/80 ag.

### Characterization of the Sialoadhesin Antigen

In order to compare the sialoadhesin molecule in developing and adult tissues, lysates from liver

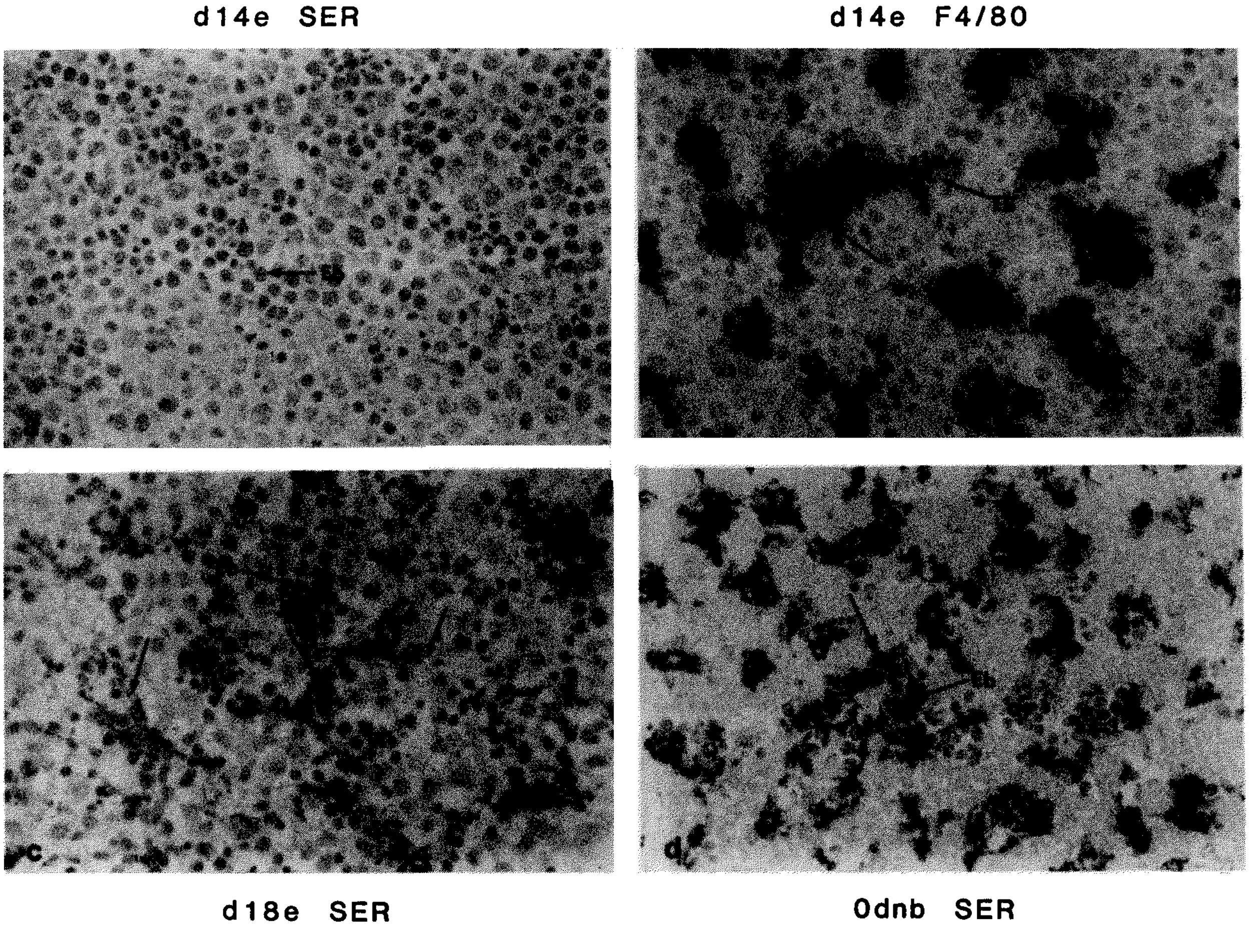

FIGURE 1. Sialoadhesin expression during liver development. Cryostat sections were stained with monospecific polyclonal antisera against sialoadhesin or F4/80 ag. Sialoadhesin was not expressed in d14 fetal liver (A) which contained numerous MØ, as shown by F4/80 ab (arrow, B). Erythroblasts (Eb) were present throughout liver and in contact with F4/80$^+$ MØ plasma membrane processes. By d18 (C), MØ expressed low levels of sialoadhesin ag (arrow) as shown by faint patchy staining, which became more intense in newborn (Odnb) mice (D). Staining with the F4/80 ab (not illustrated) showed that these cells were also F4/80$^+$. Bar=20 $\mu$m.

TABLE 1

Comparison of Sialoadhesin Induction on FLMØ and TPM[a]

| Treatment | % Mo binding >4 sheep E | |
|---|---|---|
| | FLMØ | TPM |
| Freshly isolated | 2±2 | 8±3 |
| After cultivation: | | |
| 3d 10% mouse serum | 77±15 (2) | 95±4 (0) |
| 3d HB102 (serum-free) | 67±23 (14±4) | 2±2 |

[a]2×10[6] fetal-liver cells or 2.5×10[4] TPM were plated on glass coverslips and assayed after 1–4 hr adherence or after further cultivation. Figures in parentheses show levels of rosetting in the presence of the inhibitory SER-4 mAb. Data (means±S.D.) are taken from at least two independent experiments in which 200 MØ were counted on duplicate coverslips. Results show that FLMØ acquire sialoadhesin activity whether cultured in the presence or absence of mouse serum, unlike TPM.

and spleen were examined from animals at different stages. SDS-PAGE and direct immunoblotting were performed using $^{125}$I-IgG preparations of SER-4 and 5C1, a mAb directed against the F4/80 molecule. Sialoadhesin has an apparent Mr of 185 kD under reducing conditions and 175 kD under nonreducing conditions. Western blots were performed under nonreducing conditions since reduction with β-mercaptoethanol resulted in loss of activity of both antigens. Figure 3 shows a tight band at 175 kD in lysates from d18 liver and spleen that comigrates with sialoadhesin identified previously in adult spleen. Sialoadhesin was barely detectable in lysates from d16 and earlier, which contained F4/80$^+$ MØ as indicated by the characteristic broad band at ~150 kD on $^{125}$I-5C1 blots (Fig. 3, far right). In other experiments (not shown), sialoadhesin could be detected in lysates from d17 spleen, but not liver. The nature of the ag did not change during development, although its specific activity decreased in liver as hemopoiesis declined postnatally. Control experiments carried out in the presence of excess unlabeled ab demonstrated the specificity of each procedure.

### Sialoadhesin Expression by Isolated Fetal Liver MØ

In order to confirm that d14 FLMØ lack sialoadhesin, cells were isolated by collagenase digestion and adherence to glass coverslips and, after removal of attached Eb, assayed for their ability to rosette sheep E. FLMØ did not bind sheep E (Fig. 4A and Table 1) although they retained the ability to rebind Eb via the previously described Eb receptor (EbR) (Morris et al., 1988). Immunocytochemistry with the SER-4 mAb on isolated FLMØ confirmed that these cells did not express sialoadhesin (not shown). The lack of sialoadhesin on FLMØ was not due to the isolation procedure because adult resident bone marrow MØ prepared by similar methods including collagenase digestion (Crocker and Gordon, 1986) express this receptor. FLMØ isolated between d12–d17 of gestation were all sialoadhesin-negative by erythrocyte rosetting, in agreement with the negative *in situ* immunocytochemical findings and Western blot analysis. Attempts to isolate FLMØ from later stages were unsuccessful due to poor yields and viability. Perfusion of liver with collagenase was not possible due to their small size.

Since nonstromal adult-derived MØ such as thioglycollate elicited peritoneal MØ (TPM) express high levels of SE rosetting activity after cultivation in the presence of homologous serum (Crocker, Hill, and Gordon, 1988), we asked whether sialoadhesin could be induced on FLMØ by exposure to 10% adult mouse serum for 3 days. As shown in Fig. 4B and Table 1, FLMØ cultivated in mouse serum bound large numbers of sheep E. The induced activity was completely blocked by SER-4 mAb, confirming the specificity of binding, and Western blot analysis showed that the molecule was of the correct molecular weight (Fig. 5).

Unlike induction of TPM, which required the continuous presence of mouse serum, sialoadhesin expression on cultivated FLMØ also occurred in serum-free media, although to a lesser extent than in the presence of mouse serum (Table 1). FLMØ isolated at different stages (d12–d17) were all capable of autoinduction with no apparent difference in the rates at which they acquired the antigen (not shown). FLMØ cultivated in the absence of an exogenous inducer expressed sialoadhesin ag by immunocytochemistry (not shown) and by Western blotting (Fig. 5) and ~80% of rosetting was blocked by SER-4 mAb (Table 1). In addition to MØ, fetal-liver-derived cultures prepared in serum-free medium contained flattened epithelioid cells, fibroblasts, and other unidentified cells, none of which bound sheep E. Although sialoadhesin activity was reliably detected on FLMØ cultured in the absence of mouse serum, attempts to detect an inducing activity in conditioned media obtained from these cultures gave variable results when

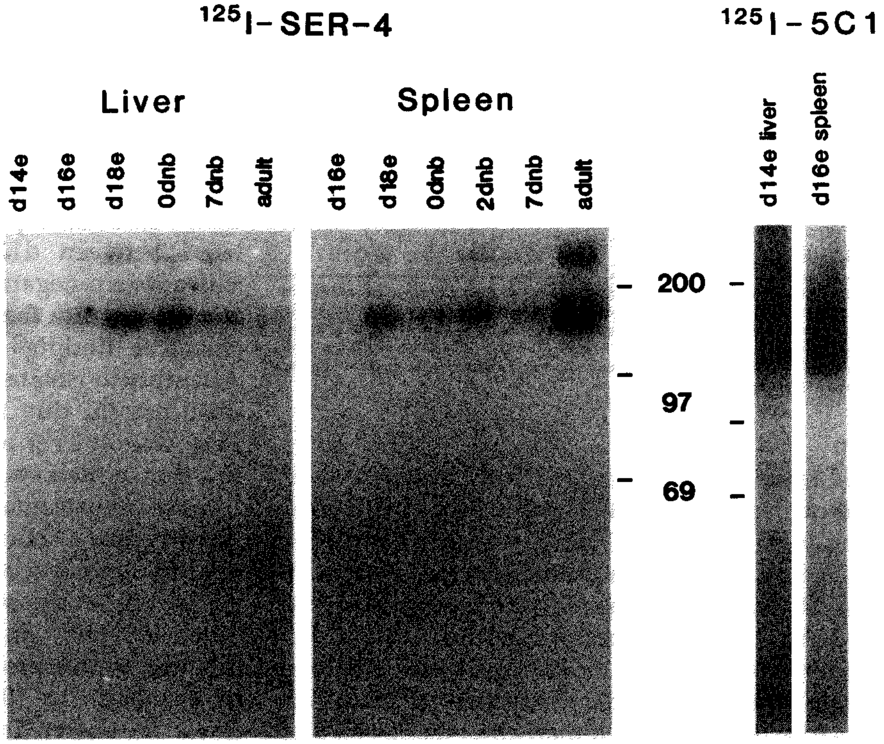

FIGURE 3. Western blots of sialoadhesin during liver and spleen development. Lysates of fetal, newborn, and adult liver and spleen were electrophoresed in 6.5% polyacrylamide gels, transferred to nitrocellulose, and probed with $^{125}$I-SER-4 or $^{125}$I 5C1 (anti-F4/80) IgG. A specific band at 175 kD in SER-4 blots can be seen in fetal liver and spleen from d18 and is identical to the ag found in adult spleen. The band above 200 represents aggregated ag. Trace levels of sialoadhesin were detected in d14 and d16 liver, and d16 spleen, although F4/80 was readily detected (far right). No bands were seen in the presence of excess corresponding unlabeled IgG (not shown). Markers show position of molecular weight standards ($\times 10^3$).

assayed on TPM. Inducing activity was detected in 5 out of 15 experiments, at a concentration equivalent to 4–11% of adult mouse serum.

### Sialoadhesin Inducing Activity in Fetal Serum

In adult mice, the expression of sialoadhesin by MØ is regulated by unidentified factor(s) that are constitutively present in normal plasma or serum, irrespective of strain or sex (Crocker, Hill, and Gordon, 1988). To test whether developing animals contain an inducing activity within their circulation, blood was collected from d13 until 2 weeks after birth and assayed on target TPM. Figure 6 shows that inducing activity was detectable in d13 fetal serum, increased during later development, and reached adult levels by 14 days after birth. Although it was difficult to determine precise levels of inducing activity, we estimate that d13 fetal serum contained ~25% and 7 dnb serum ~50% of that present in the adult, volume for volume. Control experiments with blocking mAb (SER-4) confirmed that rosetting induced by fetal and newborn serum was due to sialoadhesin (not shown). Fetal d13 serum contained 9.2 mg/ml total protein, compared with 36.6 mg/ml in the adult, indicating that the specific activity of the inducer in fetal blood was comparable to that of the adult. Finally, assays of serum taken from pregnant animals showed no differences in the level of inducer compared with nonpregnant adult mice (not shown). These studies established that inducing activity was present in the fetus at the earliest time examined, d13, before sialoadhesin expression and that levels increased with further pre- and postnatal development, in parallel with induction of receptor expression *in situ*.

### DISCUSSION

In this study, we show by immunocytochemistry, Western blotting, and rosetting analysis that sialoadhesin is not present on fetal liver MØ at the time of intense erythropoietic activity (d12–d17). Sialoadhesin is therefore not required for binding of erythroblasts to fetal liver MØ, which we showed previously was mediated via a divalent cation-dependent mechanism. Interestingly,

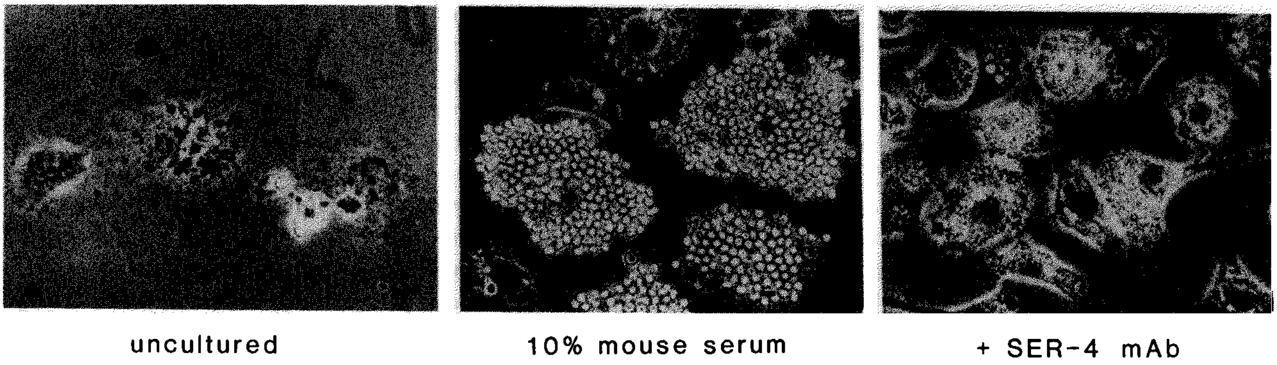

FIGURE 4. Induction of sheep E rosetting activity in cell culture. FLMØ were isolated from d14 fetal liver by adherence and assayed for sheep E binding. After removal of attached hemopoietic cells, freshly isolated MØ did not bind sheep E (A). Upon cultivation in RPMI plus 10% mouse serum for 3 days, many FL-derived MØ bound large numbers of sheep E (B). Rosetting could be completely inhibited by the SER-4 mAb (C). The refractile lipid in Fig. 4C induced by the mouse serum may obscure the fact that they are sialoadhesin-negative. Bar=10 μm.

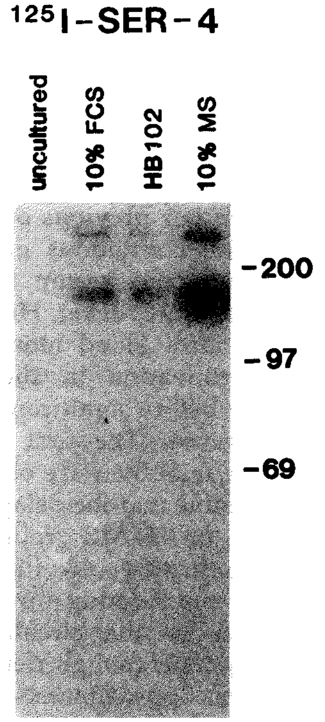

FIGURE 5. Western blots of sialoadhesin ag expression on cultured FLMØ. FLMØ were isolated from d14 fetal liver and cultured for 6 days in HB102 or 10% mouse serum. Western blot analysis was performed on lysates using $^{125}$I-SER-4 IgG. A specific band at 175 kD and aggregated ag at >200 kD can be seen in cells cultivated in the presence or absence of serum, but not in uncultured cells. Markers show position of molecular-weight standards (×10$^{3}$).

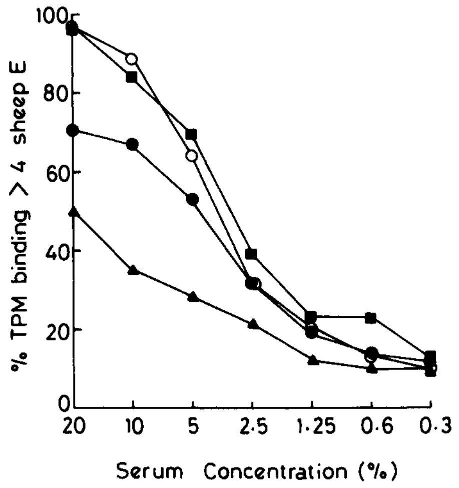

FIGURE 6. Sialoadhesin-inducing activity in serum during development. Serum was collected from fetal and newborn mice and tested for its inducing activity on TPM. d13 fetal serum (▲–▲) showed low levels of inducing activity. Repeated assays with fetal serum showed a reproducible dose response of inducer activity that increased with age (not shown). Half-maximal levels were found in 7 dnb animals (●–●) and by 2 weeks postnatally (■–■) sialoadhesin-inducing activity was comparable to that found in the adult (O–O). Values represent the mean percentages of TPM binding >four sheep E and are derived from triplicate assays. S.D. varied by less than 10% of the means and were omitted for clarity.

however, sialoadhesin is expressed on liver, spleen, and bone marrow·MØ from day 18 to birth, a stage that corresponds to the onset of myelopoiesis. Further changes in the numbers and distribution of strongly sialoadhesin-positive MØ coincided with the expansion of white pulp in spleen 1–3 weeks after birth and the development of the marginal zone. Stromal MØ therefore interact with different lymphohemopoietic cells at different stages of development. Our studies also indicate distinct functions for the two known cell-interaction receptors on stromal MØ. Although sialoadhesin and the Eb-binding receptor are expressed independently of each other on serum-induced peritoneal MØ (Crocker, Hill and Gordon, 1988) and d14 FLMØ, respectively, they are co-expressed by adult bone marrow MØ (Morris, Crocker et al., 1991). Further studies with inhibitory ab for each receptor will be required to define their individual role in binding of various ligand-bearing cells by different stromal MØ.

Our present studies provide direct evidence that sialoadhesin is not essential for fetal erythropoiesis. The situation may be different in the adult where sialoadhesin present on resident bone marrow MØ could interact with appropriate sialylated structures on erythroblasts expressed during differentiation. In adult bone marrow, developing myeloid cells are also present in MØ-hemopoietic clusters (Crocker and Gordon, 1985) and sialoadhesin is selectively concentrated within the MØ plasma membrane at contact points with immature myeloid but not erythroid cells (Crocker et al., 1990). This observation, together with the delayed expression of sialoadhesin, indicate that the function of this receptor may be more related to myeloid than to erythroid maturation. In the fetal spleen, our present studies showed that expression of sialoadhesin coincided with myelopoietic activity. However, it was not possible by immunocytochemistry at low resolution alone to establish whether fetal myeloid cells formed associations with MØ expressing sialoadhesin. Further studies involving collagenase digestion of spleen at different stages are needed to establish the presence of specific MØ–myeloid interactions during development.

Both hemagglutinin receptors are also likely to be implicated in interactions of stromal MØ and lymphoid subpopulations. In the adult, high levels of sialoadhesin are expressed on marginal metallophils in the spleen and on stromal MØ in the subcapsular sinuses and medullary cords of lymph nodes (Crocker and Gordon, 1989). These populations are closely associated with B lymphocyte subpopulations (Brelinska and Pilgrim, 1982; Fossum and Ford, 1985) and thus may represent another potential site of interaction of sialoadhesin with appropriate ligands. During ontogeny of the spleen, we observed that sialoadhesin expressing MØ were present on day 18 in the red pulp, a site of active myelopoiesis, and that developing white pulp regions were initially devoid of reactivity. The characteristic ringlike pattern of strongly labeled marginal metallophils in the inner marginal zone was first observed at approximately 7 days after birth and increased gradually to reach the complete adult pattern by four weeks. It is not possible to determine from the present studies whether these intensely sialoadhesin-expressing stellate cells are redistributed from the red pulp, which retains more weakly reactive cells, or result from induction of the receptor on preexisting or newly recruited cells which localize in this specialized microenvironment. Initial sialoadhesin expression coincides with the appearance of $IgM^+$ B lymphocytes, which are first detected in the spleen on day 17 and increase rapidly to plateau levels by 1 month of age (Towbin et al., 1979), when the sialoadhesin pattern is completed. It is therefore possible that sialoadhesin on marginal metallophils interacts with ligands on a subpopulation of B lymphocytes and plays a role in their development.

The mechanisms leading to the delay in sialoadhesin expression until d17 are unknown, but may be related to our previous observation that adult MØ require continuous exposure to (a) factor(s) in homologous serum to maintain or induce its expression (Crocker, Hill and Gordon, 1988). In vitro, adult blood monocytes require several days' cultivation in the presence of inducing activity before significant expression of receptor is observed. The first F4/80-positive monocytes in the fetal liver are observed on day 10, and it is plausible that the delay in expression of sialoadhesin on FLMØ reflects a period required for induction of its biosynthesis. Another factor contributing to the delay in expression could be the circulating level of inducing activity that, though detectable in day 13 fetal serum was approximately one-quarter of that in adult serum and did not reach adult levels until 2 weeks postnatally. FLMØ cultured in the absence of serum were able to express high levels of sialoadhesin, unlike adult-derived TPM. This suggests either that a sialoadhesin-inducing activity was produced by hepatocytes or mesenchymal cells present in these cultures, or that the FLMØ were capable of autoinduction. The *in vivo* delay of sialoadhesin expression was not mirrored in *in vitro* cultures because FLMØ from different aged fetuses showed similar rates of induction. Attempts to recover inducing activity from these cultures gave inconsistent results perhaps reflecting variable proportions of the cell type(s) responsible for its production, inconsistency in secretion, factor instability, consumption by target MØ, or the presence of inhibitors. Fetal-liver cultures represent the first *in vitro* model of serum-independent sialoadhesin induction. Further studies are required to define the source of the inducing activity within the fetus and its possible role in regulation of sialoadhesin expression and MØ function during development.

Although the ontogeny of erythroid (Metcalf and Moore, 1971) and lymphoid (Owen and Jenkinson, 1981; Verlardi and Cooper, 1984) populations are well-studied, that of MØ is virtually unexplored. The antigen and receptor markers studied here confirm that MØ are a major cell population during much of fetal life, that they play a role as stromal cells in hemopoiesis, and that their phenotype is precisely regulated during development. Our studies provide a basis for further studies to explore their functions within the immature animal.

### MATERIALS AND METHODS

### Animals

Embryos (the words embryo and fetus are used interchangeably) and newborn (nb) animals from C57B1/6 and Swiss PO (Pathology, Oxford) mating colonies were used. The presence of a vaginal plug the morning after mating was designated day (d) 0 of pregnancy and the day of birth, usually d19, as day 0 of postnatal life (Odnb). C57B1/6 and PO mice of either sex between 8–12 weeks of age were used as a source of adult material.

### Media and Reagents

RPMI-1640 was purchased from Gibco Biocult (Paisley, Scotland). The defined serum-free medium HB102 was obtained from New England Nuclear (Boston, MA). Media were supplemented with 2 mM glutamine and 20 $\mu$g/ml gentamycin. 20 mM HEPES buffer (Gibco Biocult) was routinely added to RPMI stocks. Fetal bovine serum (FBS) was purchased from Imperial Laboratories (Salisbury, Wiltshire, U.K.) and heat inactivated at 56°C for 30 min. Phosphate-buffered saline without $Ca^{++}$ and $Mg^{++}$ (PBS) was obtained from Oxoid (Basingstoke, Hampshire, U.K.). The following reagents were obtained from Sigma (Poole, Dorset, U.K.): bovine serum albumin (BSA), avidin, biotin, normal sheep serum, PMSF, EDTA, iodoacetamide, soybean trypsin inhibitor (SBTI), pepstatin, leupeptin, aprotinin, and octylglucopyranoside. Sheep erythrocytes (sheep E) were purchased from Gibco Biocult. Carrier-free Na $^{125}$I was purchased from Amersham International (Amersham, U.K.).

### Antibodies

Monoclonal antibodies (mAb): The SER-4 rat mAb recognizes sialoadhesin, a cell-interaction molecule previously termed sheep erythrocyte receptor (SER), on murine stromal tissue MØ populations (Crocker and Gordon, 1989). Two rat mAb against the F4/80 ag, specific for mature mouse MØ, were used: F4/80 (Austyn and Gordon, 1981) and 5C1, which recognizes a different epitope (prepared by P.R. Crocker and L. Turley, unpublished). SER-4 and 5C1 IgC were purified from ascites by FPLC and conventional chromatography, respectively. SER-4 and F4/80 were also used in some experiments as tissue-culture supernatants at saturation.

Polyclonal antisera: Monospecific rabbit antisera were prepared to purified spleen sialoadhesin (Crocker et al., 1991) and to F4/80 ag purified by Dr. P. Dri in our laboratory. (Lawson et al., 1990).

### Collection of Mouse Serum

Serum was collected from fetal, newborn, and adult PO mice as follows: fetal mice from d13 onwards were carefully dissected with yolk sac and placenta intact and washed in PBS. After removal of the yolk sac and placenta, fetuses were allowed to bleed from the severed umbilical vessels into a fresh sterile dish. The head was decapitated to facilitate bleeding. Fetuses from as many as five litters were collected into the same dish and care taken to avoid dilution by PBS and amniotic fluid. Newborn mice up to 1 week old were decapitated and blood collected in a sterile dish. Older newborn and adult mice were asphyxiated using $CO_2$ and blood collected by cardiac puncture.

Blood samples were allowed to clot at room temperature and serum collected after centrifugation for 5 min at 10,000 g. Protein concentrations were determined using a Bio-Rad protein assay kit (Bio-Rad Laboratories, Munich, FRG) with BSA as a standard. Serum samples were not heat inactivated before use.

### Cells

Fetal-liver cultures were prepared as described (Morris et al., 1988). Briefly, 14-day fetal livers were digested using collagenase and plated onto glass coverslips at 2x10$^6$ cells/coverslip in RPMI with 10% FBS. In order to examine SE rosetting activity on uncultured MØ, attached erythroblasts were removed from FLMØ by flushing in PBS. For continued culture, intact fetal-liver cells were left overnight in RPMI plus 10% FBS. After rinsing in RPMI, coverslips were transferred to either RPMI plus 10% mouse serum or to HB102 (serum-free medium) for 2–4 days. Thioglycollate-elicited peritoneal MØ (TPM) were obtained 4–5 days after intraperitoneal injection of 1 ml Brewer's complete thioglycollate broth and used as target cells for sialoadhesin-inducing activity (Crocker, Hill and Gordon, 1988).

### Rosetting Assays

Cells on coverslips were assayed as described (Morris et al., 1988). Rosetting assays (Crocker, Hill and Gordon, 1988) were used to determine levels of inducing activity in fetal and newborn serum and in media conditioned by fetal liver cells in culture. Briefly, 2x10$^4$ TPM were cultured in microtitre wells in RPMI plus 0.2% BSA with serial doubling dilutions of fetal and newborn sera (20% to 0.3%) or conditioned media (100% to 2.5%). A reference control of adult mouse serum (20% to 0.3%) was included. After 3 days at 37°C, sheep E were added at 0.5% v/v for 39 min. All serum samples were tested for specificity of induction by preincubating some cells with the inhibitory SER-4 mAb 30 min prior to addition of sheep E. Nonadherent sheep E were removed and the percentage of MØ binding >4 sheep E determined by phase contrast microscopy. 200 TPM were counted in triplicate wells. The percentage rosettes was plotted against log sample dilution and compared with the adult mouse reference activity.

### Immunocytochemistry

Fresh unfixed tissues from fetal, newborn, and adult C57B1/6 mice were placed in Tissue-Tek O.C.T. compound (Miles Scientific, Elkhart, IN) and snap frozen in isopentane cooled by liquid $N_2$. Yolk sac, liver, spleen, femoral bone marrow, and thymus were examined. Cryostat sections (7 $\mu$m) were fixed in acetone for 10 min at room temperature and then blocked successively with avidin (0.1 mg/ml), biotin (0.1 mg/ml), and normal sheep serum (1%) for 30 min each. Rabbit preimmune serum and antisera to sialoadhesin and F4/80 ag were diluted 1:1000 in PBS and incubated with tissue sections in a humidified chamber of 1 hr at room temperature. After extensive washing in PBS, sections were incubated with a 1:250 dilution of biotinylated sheep antirabbit IgG (Vector Laboratories, Peterborough, U.K.) in 1% mouse serum for 1 hr under the same conditions. Endogenous peroxidase activity was inhibited by incubation in 0.3% (v/v) $H_2O_2$ in methanol for 30 min. The avidin—biotin–peroxidase complex was obtained commerically and used as advised (Vectastain kit, Vector Laboratories). Ag was revealed by incubation with 0.5 mg/ml diamino-benzidine tetrahydrochloride (Polysciences, Warrington, PA), as peroxidase substrate, and 0.02% $H_2O_2$ in PBS containing 10 mM Imidazole, pH 7.4. Sections were counterstained in Mayers haematoxylin for 30 sec and mounted in DPX. Representative photographs were taken using a dark blue filter. Sections incubated with preimmune rabbit antiserum served as negative controls.

### SDS-PAGE and Western Blotting

Livers and spleens were collected from fetal, newborn, and adult PO mice, frozen in liquid $N_2$ and stored at −70°C. The earliest samples analyzed were from d14 fetal liver and d16 spleen. It was often necessary to pool organs (particularly fetal and newborn spleens) to obtain sufficient protein for analysis (for example, ~50 16-day fetal spleens for two lanes in a gel). Pooled frozen samples were weighed and placed in 1 ml of 10 mM Tris, 150 mM NaCl, pH 8, containing 2 mM PMSF, 5 mM EDTA, 5 mM iodoacetamide, 100 $\mu$g/ml SBTI, 1 $\mu$g/ml pepstatin, 0.5 $\mu$g/ml leupeptin, and 2 $\mu$g/ml aprotinin. Tissues were disrupted using a probe homogenizer (Polytron;

Kinematica GmbH, Lucerne, Switzerland) and insoluble material pelleted by ultracentrifugation at 100,000 g for 30 min. Pellets were solubilized in 100–200 $\mu$l 10 mM Tris, 150 mM NaCl, pH 8, containing 2% v/v octylglucoside and protease inhibitors (excluding SBTI). Insoluble material was removed by ultracentrifugation at 100,000 g for 30 min. Supernatants were collected and normalized for protein concentration using a Bio-Rad protein assay. Lysates (25 $\mu$g/track) were electrophoresed on 6.5% polyacrylamide gels under nonreducing conditions (Laemmli, 1970). Proteins were transferred onto nitrocellulose (Towbin et al., 1979) and probed with 0.25 $\mu$g/ml $^{125}$I-SER-4 IgG or $^{125}$I-5Cl IgG, labeled by the chloramine T method (Austyn and Gordon, 1981). Control blots for specificity were done in the presence of an 80-fold molar excess of unlabeled SER-4 or 5Cl IgG.

### ACKNOWLEDGMENTS

This paper is supported by grants from the Leukaemia Research Fund and the Medical Research Council, U.K.

*(Received May 23, 1991)*

*(Accepted May 26, 1991)*

### REFERENCES

Austyn J.M., and Gordon S. (1981). F4/80, a monoclonal antibody directed specifically against the mouse macrophage. Eur. J. Immunol. **11:** 805–815.

Brelinska R., and Pilgrim C. (1982). Significance of subcompartments of the marginal zone for direction of lymphocyte traffic within spleen pulp. Cell Tiss. Res. **226:** 155–165.

Crocker P.R., and Gordon S. (1985). Isolation and characterization of resident stromal macrophages and hematopoietic cell clusters from mouse bone marrow. J. Exp. Med. **162:** 993–1014.

Crocker P.R., and Gordon S. (1986). Properties and distribution of a lectin-like hemagglutinin differentially expressed by murine stromal tissue macrophages. J. Exp. Med. **164:** 1862–1875.

Crocker P.R., and Gordon S. (1989). Mouse macrophage hemagglutinin (sheep erythrocyte receptor) with specificity for sialylated glycoconjugates characterized by a monoclonal antibody. J. Exp. Med. **169:** 1333–1346.

Crocker P.R., Hill M., and Gordon S. (1988a). Regulation of a murine macrophage hemagglutinin (sheep erythrocyte receptor) by a species-restricted serum factor. Immunology **65:** 515–522.

Crocker P.R., Kelm S., Dubois C., Martin B., McWilliam A.S., Shotton D.M., Paulson J.C., and Gordon S. (1991). Purification and properties of sialoadhesin, a sialic acid-binding receptor of murine tissue macrophages. EMBO J. 1661–1669.

Crocker P.R., Morris L., and Gordon S. (1988b). Novel cell surface adhesion receptors involved in interactions between stromal macrophages and haemopoietic cells. J. Cell Sci. (Suppl.) **9:** 185–206.

Crocker P.R., Werb Z., Gordon S., and Bainton D.F. (1990). Ultrastructural localisation of a macrophage-restricted sialic acid binding hemagglutinin, SER, in macrophage hemopoietic cell clusters. Blood **76:** 1131–1138.

Fossum S., and Ford W.L. (1985). The organisation of cell populations within lymph nodes: Their origin, life history and functional relationships. Histopathology **9:** 469–499.

Laemmli U.K. (1970). Cleavage of structural protein during the assembly of the head of bacteriophage T4. Nature (London) **227:** 680–685.

Lawson L.J., Perry V.H., Dri P., and Gordon S. (1990). Heterogeneity in the distribution and morphology of microglia in the normal adult mouse brain. Neuroscience **39:** 151–170.

Metcalf D., and Moore M.A.S. (1971). Embryonic aspects of hemopoiesis. Front. Biol. **24:** 172–271.

Morris L., Crocker P.R., Fraser I., Hill M., and Gordon S. (1991a). Expression of a divalent cation-dependent erythroblast adhesion receptor by stromal macrophage from murine bone marrow. J. Cell Sci. **99:** 141–147.

Morris L., Crocker P.R., and Gordon S. (1988). Murine fetal liver macrophages bind developing erythroblasts by a divalent cation-dependent hemagglutinin. J. Cell Biol. **106:** 649–656.

Morris L., Graham C.F., and Gordon S. (1991b). Macrophages in hemopoietic and other tissues of the developing mouse detected by the monoclonal antibody F4/80. Development. **112:** 517–526.

Owen J.J.T., and Jenkinson E.J. (1981). Embryology of the lymphoid system. Prog. Allergy **29:** 1–34.

Towbin H., Staehelin E., and Gordon J. (1979). Electrophoretic transfer of proteins from polyacrylamide gels to nitrocellulose sheets: Procedure and some applications. Proc. Natl. Acad. Sci. USA **76:** 4350–4354.

Verlardi A., and Cooper M.D. (1984). An immunofluorescence analysis of the ontogeny of myeloid, T and B lineage cells in mouse hemopoietic tissues. J. Immunol. **133:** 672–677.

